# Sustainable Reactive Polyurethane Hot Melt Adhesives Based on Vegetable Polyols for Footwear Industry

**DOI:** 10.3390/polym14020284

**Published:** 2022-01-11

**Authors:** Maria Pilar Carbonell Blasco, María Ángeles Pérez Limiñana, Carlos Ruzafa Silvestre, Elena Orgilés Calpena, Francisca Arán Aís

**Affiliations:** Footwear Technology Centre, Campo Alto Campo, 03600 Alicante, Spain; maperez@inescop.es (M.Á.P.L.); cruzafa@inescop.es (C.R.S.); eorgiles@inescop.es (E.O.C.); aran@inescop.es (F.A.A.)

**Keywords:** biobased polymers, biobased matrix, natural feedstock, circular economy

## Abstract

The aim of this work is to develop sustainable reactive polyurethane hot melt adhesives (HMPUR) for footwear applications based on biobased polyols as renewable resources, where ma-croglycol mixtures of polyadipate of 1,4-butanediol, polypropylene and different biobased polyols were employed and further reacted with 4-4′-diphenylmethane diisocyanate. The different reactive polyurethane hot melt adhesives obtained were characterized with different experimental techniques, such as Fourier transform infrared spectroscopy (FTIR), thermogravimetric analysis (TGA), differential scanning calorimetry (DSC), softening temperature and melting viscosity. Finally, their adhesion properties were measured from T-peel tests on leather/HMPUR adhesives/SBR rubber joints in order to establish the viability of the used biobased polyols and the amount of these polyols that could be added to reactive polyurethane hot melt adhesives satisfactorily to meet the quality requirements of footwear joints. All biobased polyols and percentages added to the polyurethane adhesive formulations successfully met the quality requirements of footwear, being comparable to traditional adhesives currently used in footwear joints in terms of final strength. Therefore, these new sustainable polyurethane adhesives can be considered as suitable and sustainable alternatives to the adhesives commonly used in footwear joints.

## 1. Introduction

Sustainability is a crucial factor for the viability of the European footwear industry. The footwear and related industries are immersed in a process of transformation towards a circular economy, in which a greater use of raw materials is introduced, as well as the implementation of more efficient processes in terms of energy consumption with less environmental impact, so that they contribute to a lesser generation of waste, as well as its reuse and/or valuation [1,2].

Specifically, in the footwear sector, polyurethanes (PU) are one of the most used materials in insoles, coatings, elastomers, adhesives, etc., thanks to their great versatility. Its demand is constantly growing due to its excellent chemical, physical and mechanical properties, reaching a consumption of 18 Mt in 2019 [3]. Indeed, thermoplastic polyurethanes (TPUs) are a relevant kind of thermoplastic elastomers with a wide variety of compositions and properties [4]. Similarly, polyurethane adhesives are of great importance in the footwear sector, fulfilling technical requirements across the wide range of materials used in footwear manufacturing [3,5].

Polyurethanes are synthesized through the reaction of polyols with polyisocyanates. These polymers are characterized by the fact that their properties and application are based on their chemical nature, reaction parameters and different stoichiometric ratios (NCO/OH), chain extenders as well as the use of catalysts [6]. In addition, one of the most environmentally friendly types of polyurethane adhesives are reactive polyurethane hot melt adhesives (HMPUR). They are a type of low molecular weight terminal isocyanate prepolymer prepared from polyester and polyether polyols and a diisocyanate [7]. The HMPUR adhesives can cure with moisture and form a cross-linked structure after application, giving them superior properties in thermal stability, solvent resistance as well as good aging resistance, which are better than those of hot melt adhesives [8]. These adhesives are solid prepolymers with a lower melting point than conventional hot melts, and are applied at a low temperature of 90 to 140 °C, flowing well and wetting the surface where they are applied. HMPUR acts in two distinct phases as moisture curing adhesives, i.e., in a first phase known as physical crosslinking, developing green strength as a result of the cooling process similar to conventional hot melt. In the second phase, also known as chemical crosslinking, the isocyanic groups begin to react with the moisture and/or the bonded materials, producing an increase in molecular weight resulting in a fully reacted polymer after the cure time has elapsed. This two-stage reaction system allows a higher reaction strength and stronger, more durable bonds than conventional melts [9]. Moreover, these adhesives are monocomponent and they are applied to one side. Therefore, HMPURs are good candidates for use in the footwear industry thanks to their versatility, durability and good mechanical properties.

There is recent literature on the development of VOC-free solvent polyurethane adhesive from vegetable oil, which proved to be less toxic, reducing environmental pollution and risk to human health. These studies also resulted in different types of vegetable oil-based polyurethane adhesives that can replace the petroleum-based one [10].

There are many alternative ways to obtain polyurethane from renewable raw materials, such as vegetable oil (soya, sunflower, etc.). In recent years, vegetal oils are one of the main alternatives to stop using petroleum-derived materials to produce polyols. These chemical substances are one of the basic raw materials for the development of polyurethane [10]. The recent alternatives used as polymer synthesis reagents have many advantages, such as their biodegradability, low toxicity, sustainability, industrial feasibility, cost competitiveness and the design of final polymer properties. In recent years, many studies have allowed alternative fuel oils as a raw material base. For instance, soybean, sunflower, rapeseed, linseed and castor oil are the most used oils to synthesize polymers through chemical modification [11,12].

Several studies have been discussed about the development of vegetal oil-based PU adhesive from modified polyols using epoxidized soybean oil, canola oil and palm oil with alcohols and acid [13,14,15,16]. The main component of vegetable oil is triglyceride, which is an ester of glycerol and three long-chain fatty acids [17]. Fatty acids can be obtained from these vegetable oils by hydrolysis, and then converted into polyols through different production methods (epoxidation, ozone decomposition, etc.).

The use of biobased polyols instead of polyols from non-renewable resources results in higher green carbon content in polyurethane adhesives. In the last few years, the tremendous growth of biobased polyols, such as ester-based polyols, has resulted in a new view of the properties of polyurethanes, even though the chemical composition of these biopolyols is the same as petrochemicals [18].

Suzuki et al. and Gogoi et al. [19,20] studied traditional ester-based polyurethanes and the effect of the [NCO]/[OH] ratio on their gelation process, network structure, microphase separation, morphology, rheological properties and thermal properties. These polyols are characterized by higher viscosity depending largely on the molecular mass of the polyol, being an important aspect together with the number of hydroxyls and acids in the polyurethane production. In the literature, there are works describing selected properties of biobased thermoplastic polyurethanes obtained with biobased polyester polyol.

Considering the influence of reagents on the properties of polyurethanes, attention has been paid to polyols, being one of the major components in weight percentage of each polyurethane. Depending on the chemical structure (polyester, polyether, hybrid polyols), topology (linear or branched), average molecular mass and functionality, different types of polyols can be used to obtain various types of PUs. Nowadays, it is necessary to design biobased polyols characterized by selected properties suitable for PU synthesis [21,22,23].

This work is focused on the synthesis and characterization of reactive polyurethane hot melt bioadhesives, which are based on polyols derived from renewable resources, allowing the reduction or removal of fossil raw materials and selection of eco-friendly ones. These sustainable raw materials provide polyurethane adhesives with additional beneficial non-toxic and sustainable properties without compromising their performance during their useful life. For this purpose, three polyester polymers from renewable sources were selected, adhesives were synthesized with different proportions of the selected polyols and MDI was used as isocyanate. The properties of the new adhesives were evaluated with experimental techniques, such as DSC, TGA, FTIR, softening temperature and melting viscosity. Finally, their adhesion properties were measured from T-peel tests on leather/HMPUR adhesives/SBR rubber joints.

## 2. Materials

Reactive polyurethane hot melt adhesives were synthesized from several macroglycol mixtures of polyadipate of 1,4-butanediol (Hoopol F-580, Synthesia Technology, Barcelona, Spain) [24], with an average molecular weight (M_w_) of 2826 g·mol^−1^ polypropylene glycol (PPG) (Quimidroga SA. Barcelona, Spain) (M_w_ = 425 g·mol^−1^) and three different biobased polyester polyols (DINACOLL TERRA, Evonik, Germany [25]); the properties of these polyols are included in Table 1. In addition, MDI, 4-4′methylene diphenyl diisocyanate (Sigma-Aldrich, Barcelona, Spain) was used as a diisocyanate as it is solid, and it is the best performing to produce polyurethane adhesives with the appropriate characteristics for the intended application at present.

### 2.1. Synthesis of Polyurethanes for Reactive Hot Melt Adhesives (HMPUR)

**Figure 1:**
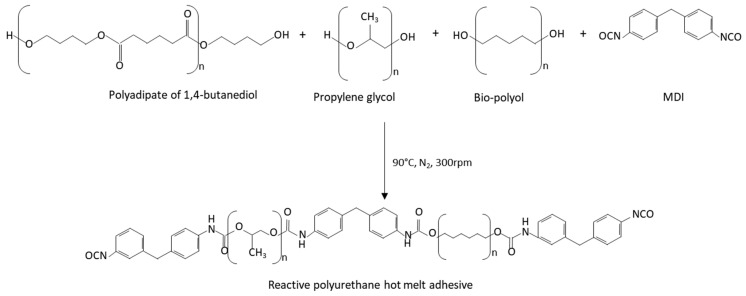
Scheme of the synthesis of the reactive polyurethane hot melt adhesives.

Reactive polyurethane hot melt adhesives were synthesized by the prepolymer method (Figure 1), using an optimal NCO/OH index of 1.5. They were synthesized under a nitrogen atmosphere at 90 °C in a glass vessel placed in an oil bath and equipped with a mechanical stirrer (Heidolph RZR 2021, Kelheim, Germany) providing a 300 rpm agitation with a stainless-steel rod attached to the stirrer to mix the reagents, according to previous research carried out by the authors [26,27,28].

The referenced reactive polyurethane hot melt adhesive (PUREF) was obtained by equal mixing of polyadipate of 1,4-butanediol and propylene glycol. For the incorporation of the different polyols (T), the propylene glycol (PPG) was partially replaced in the formula according to Table 2.

The percentage of free NCO was calculated applying the *n*-dibutylamine titration method [29]. Once the desired index was reached, the reaction took place. Then, the HMPUR adhesive obtained was stored in hermetic disposable cartridges to be applied later by a manual hot melt applicator and further characterization.

### 2.2. Characterization of the Reactive Polyurethane Hot Melt Adhesives

#### 2.2.1. Softening Temperature

The determination of the softening temperature of the HMPUR was assessed according to EN1238 [30]. The softening temperature indicates the temperature above which the viscous properties of the adhesive become dominant. The standard test defines the softening temperature as the temperature at which a steel ball passes through a ring filled with adhesive while immersed in a water, glycerol or mineral oil bath (depending on the softening temperature).

#### 2.2.2. Melting Viscosity

The apparent viscosity of the reactive polyurethane hot melt adhesive was evaluated using a Brookfield Thermosel viscometer equipped with an SC4-27 spindle according to the test procedure described in the ASTM D3236-15 standard [31]. The measurement of the viscosity consists of a thermostatized camera which melts the adhesive, allowing the viscosity to be measured at a controlled temperature. This value determines the ease or difficulty of the application of the adhesive to the substrate, the working temperature, the open time, etc.

#### 2.2.3. Fourier Transform Infrared Spectroscopy (FTIR)

The chemical properties of the HMPUR adhesive were determined using a Varian 660-IR infrared spectrophotometer (Varian Australia PTY LTD; Mulgrave, Australia). Attenuated total reflection (ATR) technology was used to perform 16 scans at a resolution of 4 cm^−1^.

#### 2.2.4. Thermogravimetric Analysis (TGA)

The thermal stability of HMPUR was performed using a TGA 2 STARe System thermal balance equipped with STARe software (Mettler-Toledo, Switzerland). Approximately 7 to 10 mg of adhesive sample was placed in an alumina crucible. The sample was heated from 30 to 600 °C at 10 °C/min in an inert nitrogen atmosphere (flow rate = 30 mL min^−1^).

#### 2.2.5. Differential Scanning Calorimetry (DSC)

The thermal properties of polyurethane adhesives were studied using a DSC3 + STARe Systems calorimeter (Mettler-Toledo AG, Schwerzenbach, Switzerland). Samples of approximately 9–12 mg in an aluminum pan were employed. The experiments were conducted in an inert nitrogen atmosphere (flow rate = 30 mL min^−1^) at a heating or cooling rate of 10 °C/ min. Two consecutive runs were completed: (i) heating from −15 to 110 °C, then isothermally heating at 110 °C for 3 min to eliminate the thermal history of the sample; (ii) the second heating from −65 °C to 100 °C, followed by isothermal cooling at 25 °C for 45 min. The optimal conditions for DSC experiments were previously optimized by the author in a previous work [32].

#### 2.2.6. T-peel Strength Test

Adhesion properties were evaluated according to the procedure described in the standard EN 1392:2007 [33]. Standard materials were used as a soling and upper material to be joined: vulcanized rubber SBR-2 and a chrome-tanned split leather, respectively. The split leather was provided by the Spanish company “Palomares Piel, S.L” (Elda, Spain). In this way, the bondability of HMPURs was evaluated in split leather/SBR rubber joints.

Before joint formation, each adherent was duly treated. Split leather test samples were roughened at 2800 rpm with a P100 aluminum oxide abrasive cloth (Due Emme Abrasivi, Pavia, Italy) using a roughing machine (Superlema S.A., Zaragoza, Spain). The SBR-2 rubber was roughened and halogenated with 2 wt% trichloroisocyanuric acid solutions in ethyl acetate. Both are a typical treatment in the footwear industry today. The following procedure was used to prepare adhesive bonded joints of 150 × 30 mm (SBR rubber/HMPUR/leather).

Joint formation was carried out after 30 min of the adhesive application. To enhance contact between both adhesive films, the substrates were activated by infrared radiation at 80 °C in a CAN 02/01 temperature-controlled heater provided by AC&N (Elda, Spain). The strips were immediately placed in contact with each other and a pressure of 1.8 bar was applied for 10 s to achieve a suitable joint. Then, adhesive joints were stored at 23 °C and 50% relative humidity for 72 h. Finally, T-peel strength measurement was performed in an Instron 1011 universal testing machine (Instron Ltd., Buckinghamshire, UK) at a crosshead speed of 100 mm/min. The bond strength values were then calculated as a function of the piece width, strength value and scale obtained with the testing machine, thus obtaining an average and typical deviation of the five values for each sample.

## 3. Results

This section will provide a concise description of the experimental results, their interpretation as well as the experimental conclusions that can be drawn.

### 3.1. FTIR Analysis

**Figure 2:**
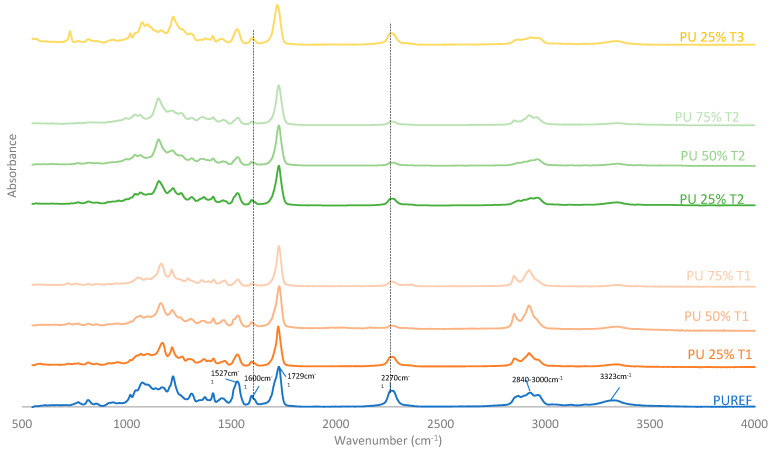
FTIR spectra of the HMPUR adhesives based on different biobased polyols (T1, T2 and T3, respectively), and different content of biobased polyols.

FTIR was performed to further analyze the chemical structures of biobased HMPUR adhesives synthetized with different amount and bio-polyols before curing. Figure 2 shows the FTIR spectra of the HMPUR adhesives with biobased polyols, as well as the spectrum corresponding to the conventional HMPUR adhesive. FTIR spectra show the characteristic bands of the polyurethane prepolymer adhesives and the band corresponding to the free reactive isocyanate group. The reactive polyurethane adhesive obtained requires an excess of isocyanate to react with moisture and subsequently polymerize or chemically cure. In addition, it is worth noting the following tapes appearing on all HMPUR adhesives: NH elastic tape at 3324 cm^−1^, CH elastic tape at 2840–3000 cm^−1^, *st* C=O of ester and urethane. The ester group is at 1729 cm^−1^, the C=C band of the aromatic group is stretched at 1600 cm^−1^, the CN and δ NH bands are stretched at 1527 cm^−1^ and the bands stretched at 2270 cm^−1^ correspond to the extended reactive isocyanate groups [34,35]. In adhesives with polyols obtained from renewable sources, there is a decrease in free isocyanate content, with the decrease being more marked when the amount of biobased polyol is higher. The aromatic group (1600 cm^−1^) and the 1527 cm^−1^ have the same behavior, increasing with the amount of biopolyol used, regardless of the type of polyol. These results indicate that the biobased HMPUR adhesives were successfully synthesized using different polyols and different amount of polyols as macroglycols used.

### 3.2. Thermal and Physical Properties of Biobased HMPUR Adhesives

**Figure 3:**
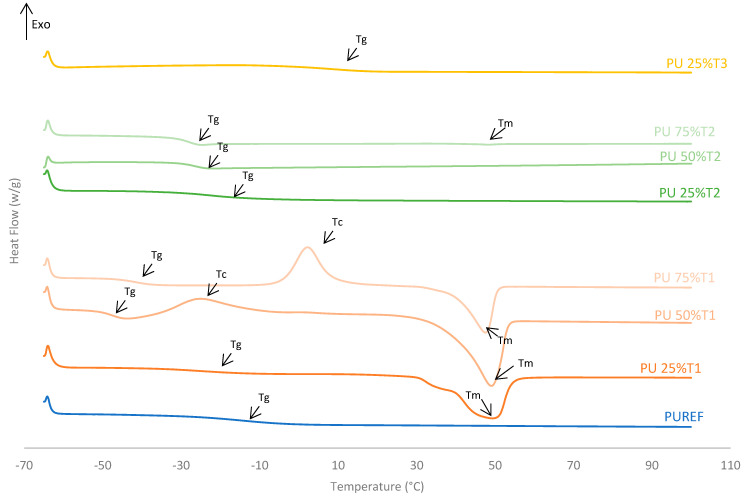
DSC thermograms of the HMPUR adhesives with different biobased polyols (T1, T2 and T3, respectively), and different content of biobased polyols. Second heating run.

The thermal properties of the biobased HMPUR adhesives prepared with bio-polyols at different amounts were analyzed using differential scanning calorimetry (DSC). Figure 3 shows the DSC thermograms corresponding to the second heating run, and Table 3 illustrates the parameters obtained from these thermograms. The conventional urethane prepolymer adhesive (PUREF) did not show the crystallization and melting process of the soft segments, but it did show a glass transition temperature (T_g_) around −14.2 °C due to soft segments.

The addition of polyol T1 changes the structure of HMPUR adhesives, since the glass transition temperature decreases when a higher amount of polyol 1 is incorporated. T1 polyol 1 also imparts cold crystallization to the HMPUR adhesives due to a rearrangement of the soft segments. The higher the amount of T1 polyol used is, the higher the cold crystallization temperature is, and thus, the higher the enthalpy of crystallization, due to the crystalline nature of the biobased polyol (Figure 3, Table 3). Finally, an endothermic peak corresponding to the melting process of the crystalline fraction of the soft segments appeared (melting temperature T_m_). When part of the PPG is replaced by the polyols T2 and T3, respectively, the behavior of the HMPUR adhesives is very similar to that of the reference adhesives, according to the similarity of the polyols with the conventional polyol. The incorporation of polyol T2 causes the glass transition temperatures to move to lower temperatures, and when the higher amount of polyol is incorporated, the glass transition temperature lowers. This is possibly due to a decrease in amorphous chains with increasing amount of polyol T2, leading to more phase separation in the structure of the HMPUR adhesives [36,37,38].

Moreover, the incorporation of polyol T3 produces an increase in the glass transition temperature (T_g_) since polyol T3 has an amorphous character. Moreover, these adhesives have no melting point, this indicates that their structures are not crystalline, and this is related to the fact that they increase their viscosity (Table 3).

**Figure 4:**
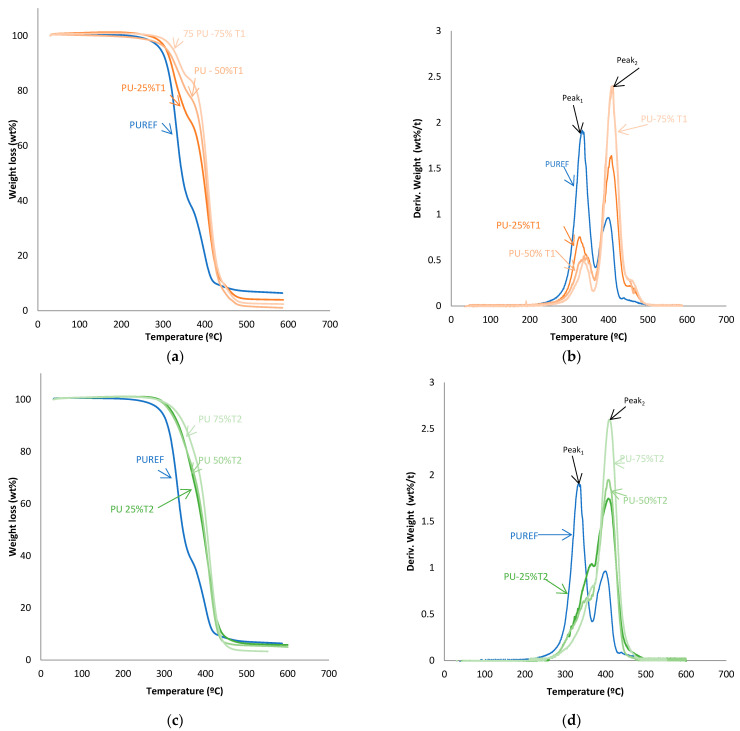

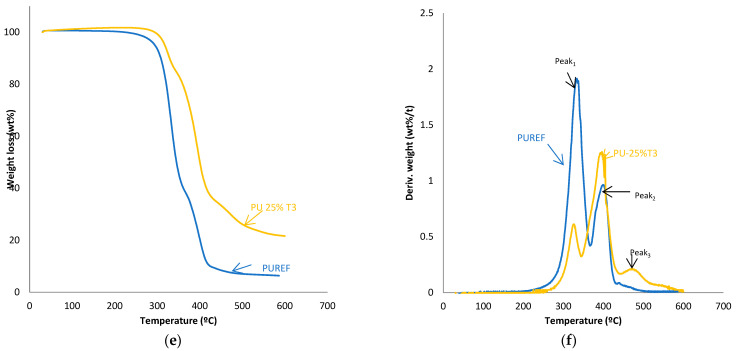
TG and DTA curves of the HMPUR with different biobased polyols, T1 (**a**,**b**), T2 (**c**,**d**) and T3 (**e**,**f**), respectively, and different content of biobased polyols.

The thermal stability of the HMPUR adhesives was evaluated with thermogravimetric analysis. Figure 4 shows TGA and derivate thermogravimetric analysis (DTG) curves of the HMPUR adhesives with different content of T1, T2 and T3 biobased polyols, respectively. The temperatures and weight loss of the different HMPUR adhesives decompositions are provided in Table 4. According to the thermograms, HMPUR adhesives synthesized with T1 and T2 polyols and decompose apparently in two stages, occurring around 330 °C and 400 °C, respectively, as the reference HMPUR adhesive [18]. The first stage of degradation is related to carbonate bonds, whereas the second stage originates from the polyol structure. Partial substitution of the conventional polyol by the different T1 and T2 polyols introduces changes in the thermal stability of the adhesives, increasing as the degree of substitution grows. The first decomposition corresponds to the soft segments, and there is a decrease in the amount of weight loss when more biopolyol is used. On the contrary, in the second decomposition, there is an increase in the loss of mass with the higher amount of biopolyol used, as a consequence of the formation of new hard segments between the biopolyols and the diisocyante. In both cases, the trend is the same for both T1 and T2 biopolyols. When the T3 polyol is used, only the small amount incorporated produces three decompositions in the new adhesive (PU-25%T3) due to the amorphous nature of the polyol.

### 3.3. Adhesion Performances of Biobased HMPUR

The melting viscosity and the soft temperature of the HMPUR synthesized are included in Table 5.

The viscosity of HMPUR adhesives is increased by the incorporation of the biobased polyol in all cases; the more propylene glycol replaced by biobased polyols, the higher the viscosity of the HMPUR adhesives is. These changes could be attributed to an increase in the molecular weight of the polyurethane. The final application of these adhesives is not affected by the increase in viscosity, so all adhesives flow properly.

Moreover, biobased HMPUR adhesives have a softening point similar to the conventional HMPUR adhesive at around 60 °C. In the case of biobased HMPUR adhesives, they present a content of 25% T3 biobased polyol and have a higher softening point as conventional HMPUR adhesive.

**Figure 5:**
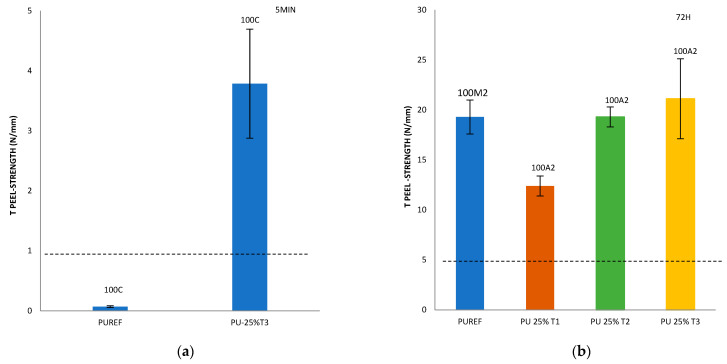

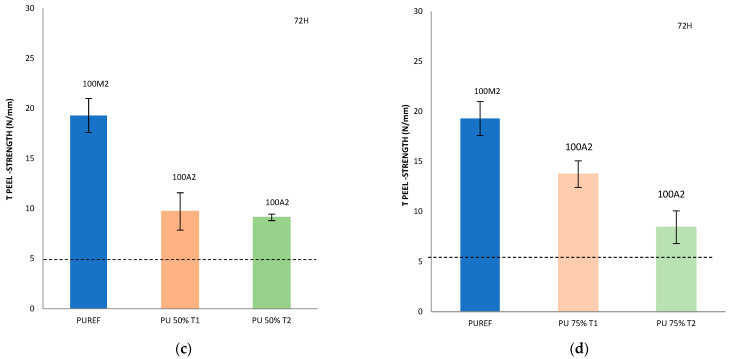
T-peel strength values of leather/biobased HMPUR adhesive/SBR joints after 5 min (**a**) and 72 h (**b**–**d**). Locus failure: C: cohesive failure in the adhesive, with threads; M2: rubber cohesive failure; A2: adhesive failure to the SBR2 rubber.

Finally, with the aim of analyzing the effect of the addition of different amounts of T1, T2 and T3 biobased polyols on the final adhesive strength of the polyurethanes, a T-peel test on the leather/SBR rubber joints was carried out (Figure 5). According to the standard EN 15307 [39], all percentages of T1, T2 and T3 biobased polyols added to the HMPUR adhesives successfully meet the final quality requirements of footwear. The initial peel resistance (after 5 min) required must be ≥1 N/mm and the final adhesion strength must be ≥2.5 N/mm for low stress bonds (e.g., in infant, indoor and fashion footwear) [24] and ≥5.0 N/mm for very high stress upper-sole bonds, such as in mountain footwear.

In addition, it is important to consider not only the peel resistance value, but also the way the separation occurs, that is, the appearance of the separation surface. Generally speaking, adhesion failure may be an adhesion failure or a cohesive failure, or a combination of the two. Adhesive failure (A) occurs in the interfacial adhesion between the adhesive and the substrate. By visual inspection, it can be observed that the adhesive remains on one of the two substrates after separation. More specifically, A2 represents the failure of adhesion of SBR rubber. In addition, cohesive failure can be of two types: adhesive cohesive failure (C) or base material cohesive failure (M), where M2 is the cohesive failure of SBR rubber, leading to the tearing of this material [28,40,41,42,43,44].

Compared with the reference adhesive (PUREF), the biobased polyols of SBR2 rubber partially replace the propylene glycol polyols, and the adhesion value of the adhesive will be reduced. In addition, since the reference adhesive has rubber cohesive failure, all biobased HMPUR adhesives share the same adhesive failure of the SBR2 rubber, so the failure has changed. According to the standard tests, all biobased HMPUR adhesives meet the minimum quality requirements necessary for footwear applications. The decrease in the adhesion values is due to the reduction in the number of soft segments achieved by the introduction of T1 and T2 biopolyols, respectively.

By incorporating T3 polyol, initial adhesion is obtained, meeting the specific requirements in footwear. This phenomenon does not occur in the reference adhesive nor with the incorporation of T1 and T2 polyols in different amounts.

Furthermore, the incorporation of 25% T3 polyol increases the adhesion 72 h after bonding. This is probably due to the new hard segments generated by the amorphous nature of the T3 polyol.

## 4. Conclusions

We are at a decisive turning point that calls for quick transformation to ensure a brighter, more sustainable future. The partial replacement of petroleum-based polyols by biobased polyols to obtain more sustainable adhesives from renewable sources with similar performance to traditional adhesives is already feasible, thus contributing to the carbon-neutrality of the European industry. Modified HMPUR adhesives, with different biobased polyols, can be prepared using the prepolymer method. Examination of their structures, thermal properties and viscosity properties revealed that the change of the bio-polyols did not affect the chemical structure but the thermal structure.

FT-IR results indicated that partial substitution of PPG by biobased polyols do not change the chemical composition of polyurethanes, regardless of the amount and nature of polyols. This makes it possible to obtain biobased HMPUR adhesives with the same chemical composition as conventional adhesives.

The incorporation of the T1 polyol with crystalline structure imparts cold crystallization to the new polurethanes, resulting in an increase in temperature as their content increases. When part of the PPG is replaced by the polyols T2 and T3, respectively, the behavior of the HMPUR adhesives is very similar to that of the reference adhesives according to the similarity of the polyols with the conventional polyol.

Thermal stability increases with the incorporation of the biobased polyols. T3 polyol produces three decompositions in the new bioadhesive of the polyurethane adhesive due to the amorphous nature of polyurethane.

The T-peel strength of the synthesized adhesives greatly exceeded the minimum strength values required for the most demanding upper to sole joints, according to the quality requirements of the European standard EN 15307. All the adhesives obtained have high strengths and all of them could be used for final performance. In particular, the adhesive containing 25% polyol T3 imparts green adhesion, thanks to its amorphous structure. This adhesion is not obtained with other bio-polyols. This is a major breakthrough for use in the footwear sector, as the initial adhesion will shorten the drying time of the joints.

The incorporation of different types of biobased polyols, with a maximum amount of 75% of biobased polyols, allows the production of HMPUR adhesives with different properties and with adhesion values that comply with the recommendations for the footwear industry. The new HMPURs offer several advantages due to their cross-linked structure, implying adequate strength and excellent adhesion to various materials.

These bio HMPUR adhesives can be used in the footwear industry, as they combine the advantages of reactive and hot-melt adhesives. They are 100% solids and do not need drying time to allow solvents to evaporate, as they are solids and are monocompound adhesives so they do not need additional crosslinking, thus avoiding the handling of hazardous chemicals by the users. Moreover, they allow the automation or robotization of the process. These results showed that a wide range of reactive polyurethane adhesives can be obtained with these commercially available biopolyols, and could interestingly improve the sustainability of polyurethanes for various applications and in particular in the footwear sector.

Finally, these new adhesives open a new way to contribute to sustainability and climate neutrality of the products where they are applied, particularly in footwear production. They also contribute to the sustainable development of the European footwear industry, thereby contributing to the achievement of the sustainable development goals (SDGs), in particular SDG 12 and 13, responsible consumption and production, and climate action. Finally, with SDG 17 because without technological cooperation all the other objectives cannot be achieved.

## Figures and Tables

**Table 1:** Properties of biobased polyester polyols.

Polyol	Chemical Description	Theoretical Hydroxyl Index, I_OH_	M_w_	Functionality	Renewable Carbon%
T1	Polyester polyol, crystalline and saturated solid	35	3500	≈2	100
T2	Polyester polyol, liquid saturated	33	3500	≈2	>85
T3	Polyester polyol saturated and amorphous solid	50	2200	≈2	>30

**Table 2:** Nomenclature and chemical composition of the synthesized polyurethanes.

HMPUR Adhesives	Propylene Glycol (wt%)	Biobased Polyol (wt%)	Diisocyanate
PUREF	100	-	4-4’methylenediphenil diisocyanate (MDI)
PU-25%T1	75	25	
PU-50%T1	50	50	
PU-75%T1	25	75	
PU-25%T2	75	25	
PU-50%T2	50	50	
PU-75%T2	25	75	
PU-25%T3	75	25	

wt% with respect to the stoichiometric quantity of polyol employed.

**Table 3:** DSC results of HMPUR adhesives calculated from the 2nd heating run.

HMPUR Adhesives	T_g_ (°C)	T_c_ (°C)	∆H_c_ (J/g)	T_m_ (°C)	∆H_m_ (J/g)
PUREF	−14.2				
PU-25%T1	−25.0			49.3	−35.9
PU-50%T1	−47.5	−25.3	17.9	49.1	−45.4
PU-75%T1	−41.5	2.2	20.2	47.5	−22.6
PU-25%T2	−23.9				
PU-50%T2	−26.3				
PU-75%T2	−27.9			48.11	−0.4
PU-25%T3	8.6				

**Table 4:** TGA results of biobased HMPUR adhesives.

HMPUR Adhesives	T_1_ (°C)	Weight Loss_1_ (%)	T_2_ (°C)	Weight Loss_2_ (%)	T_3_ (°C)	Weight Loss_3_ (%)	Residue (%)
PUREF	333.5	61.32	398.9	29.21	-	-	8.37
PU-25%T1	327.4	30.48	407.4	62.76	-	-	5.35
PU-50%T1	337.7	21.89	410.8	75.15	-	-	1.59
PU-75%T1	338.1	15.91	409.1	79.54	-	-	3.09
PU-25%T2	366.5	33.23	407.3	59.54	-	-	7.08
PU-50%T2	373.5	29.43	407.2	63.18	-	-	5.84
PU-75%T2	375.5	22.07	409.8	73.43	-	-	3.78
PU-25%T3	326.0	19.07	396.3	45.99	472.3	8.54	26.28

**Table 5:** Melting viscosity and softening point of the biobased HMPUR adhesives.

HMPUR Adhesives	Viscosity (mPa·s) T= 120 °C	Softening Point (°C)
PUREF	10,000	58
PU-25%T1	10,000	59
PU-50%T1	18,500	63
PU-75%T1	29,500	62
PU-25%T2	13,000	59
PU-50%T2	52,500	60
PU-75%T2	28,500	55
PU-25%T3	12,500	68

## Data Availability

The data presented in this study are available upon request from the corresponding author.
